# Somatic Mutations of *PIK3R1* Promote Gliomagenesis

**DOI:** 10.1371/journal.pone.0049466

**Published:** 2012-11-14

**Authors:** Steven N. Quayle, Jennifer Y. Lee, Lydia W T. Cheung, Li Ding, Ruprecht Wiedemeyer, Robert W. Dewan, Emmet Huang-Hobbs, Li Zhuang, Richard K. Wilson, Keith L. Ligon, Gordon B. Mills, Lewis C. Cantley, Lynda Chin

**Affiliations:** 1 Department of Medical Oncology, Dana-Farber Cancer Institute, Boston, Massachusetts, United States of America; 2 Center for Molecular Oncologic Pathology, Dana-Farber Cancer Institute, Boston, Massachusetts, United States of America; 3 Division of Signal Transduction, Cancer Center, Beth Israel Deaconess Medical Center, Boston, Massachusetts, United States of America; 4 Department of Systems Biology, Harvard Medical School, Boston, Massachusetts, United States of America; 5 Department of Systems Biology, The University of Texas M.D. Anderson Cancer Center, Houston, Texas, United States of America; 6 The Genome Institute, Washington University School of Medicine, St Louis, Missouri, United States of America; 7 Department of Genetics, Washington University School of Medicine, St Louis, Missouri, United States of America; University of Michigan School of Medicine, United States of America

## Abstract

The phosphoinositide 3-kinase (PI3K) pathway is targeted for frequent alteration in glioblastoma (GBM) and is one of the core GBM pathways defined by The Cancer Genome Atlas. Somatic mutations of *PIK3R1* are observed in multiple tumor types, but the tumorigenic activity of these mutations has not been demonstrated in GBM. We show here that somatic mutations in the iSH2 domain of *PIK3R1* act as oncogenic driver events. Specifically, introduction of a subset of the mutations identified in human GBM, in the nSH2 and iSH2 domains, increases signaling through the PI3K pathway and promotes tumorigenesis of primary normal human astrocytes in an orthotopic xenograft model. Furthermore, we show that cells that are dependent on mutant P85α-mediated PI3K signaling exhibit increased sensitivity to a small molecule inhibitor of AKT. Together, these results suggest that GBM patients whose tumors carry mutant *PIK3R1* alleles may benefit from treatment with inhibitors of AKT.

## Introduction

Glioblastoma (GBM) represents the most advanced and aggressive form of glioma, with current standard of care providing patients with a median survival of approximately 14 months [1]. Recent efforts to comprehensively characterize the genomes of primary GBM's have established that this disease is driven by numerous and diverse genetic events in individual patients [2], [3]. However, integrative global network analyses of these datasets identified three core networks that were simultaneously altered in the majority of patients [2], [3]. The phosphoinositide 3-kinase (PI3K) pathway was one of the most frequently targeted signaling pathways in these analyses, with recurrent genetic alterations found at multiple key nodes in the PI3K signaling cascade.

The PI3K pathway plays a critical role in regulating cellular responses to growth factors and other environmental cues. The Class IA PI3K heterodimer, which is composed of a P110 catalytic subunit and a P85 regulatory subunit, is activated upon association of the P85 subunit with upstream adaptor proteins or receptor tyrosine kinases [4]. These interactions relieve the inhibitory activity of P85 on P110, thus allowing P110 to phosphorylate its lipid substrates and subsequently induce activation of downstream effector molecules. Dominant activating mutations of *PIK3CA*, encoding P110α, have been identified in a variety of tumor types, including GBM, breast, and colon cancer, and have been shown to promote activation of P110α despite the presence of the P85 negative regulatory subunit [2], [5]–[7]. Here, we demonstrate that somatic mutations in the iSH2 domain of *PIK3R1*, encoding P85α, also promote GBM tumorigenesis and provide an independent mechanism by which tumors deregulate the PI3K signaling cascade.

## Materials and Methods

### Plasmids and mutagenesis

Flag-tagged human *PIK3R1* was provided by Dr. J. Stommel (National Cancer Institute, Bethesda, MD). Somatic mutations of *PIK3R1* identified in GBM by The Cancer Genome Atlas (TCGA) were engineered into this construct using site-directed mutagenesis, and all resulting constructs, as well as wildtype Bovine P110α-HA, were cloned into the pLenti4/V5-DEST lentiviral expression vector (Invitrogen).

### Cell culture and transformation assays

All cell lines were maintained in DMEM (Invitrogen) supplemented with 10% fetal bovine serum (FBS; Cell Generation) and 1% penicillin/streptomycin unless otherwise noted. Anchorage-independent growth was assessed by seeding triplicate wells of 6-well plates with 10,000 cells per well in 0.4% agarose on top of a bottom layer of 0.7% agarose. Upon the formation of colonies, soft agar plates were stained with Iodonitrotetrazolium chloride (Sigma) and the colonies were counted manually.

### *In vivo* tumorigenicity assays

10^6^ cells were mixed with 50% Matrigel (Fisher) and injected subcutaneously into flanks of 4–6 week-old NCR Nude mice (Taconic) and monitored for tumor development. Upon appearance of a tumor, weekly tumor measurements were taken and total tumor volume was estimated by the equation *l*·*w*·*h*·π/6 (*l* = length, *w* = width, *h* = height). Alternatively, 5×10^5^ cells were suspended in 3 µL Hanks Balanced Salt Solution and injected intracranially in NCR Nude mice. Briefly, mice were anaesthetized with 100 mg/kg Ketamine and 10 mg/kg Xylazine and placed in a stereotactic frame using ear bars. A hole was bored 1 mm anterior and 2 mm lateral to Bregma, and 3 µL of cell suspension was injected at a depth of 3 mm using a 30G Hamilton syringe. The incision was closed with a surgical clip and the mice were monitored for the development of neurological or physical symptoms. Upon onset of neurological symptoms, or upon termination of the experiment, tumors were harvested and processed for pathological and molecular analyses. All animal experiments were approved by Harvard's Institutional Animal Care and Use Committee (IACUC) under Protocol No. 04-136.

### *In vitro* kinase assay

293T cells were co-infected with wildtype P110α and either wildtype or mutant P85α constructs. Cell lysates were generated by scraping cells in ice-cold RIPA buffer (50 mM Tris-HCl pH 7.4, 150 mM NaCl, 1% NP-40, 0.5% deoxycholate, 0.1% SDS, Complete Mini Protease Inhibitor Cocktail [Roche], and Phosphatase Inhibitor Cocktail Set I and II [Calbiochem]). Immunoprecipitation of P85α complexes was performed using anti-Flag (M2) antibody (Sigma) and expression/interaction of the transgenes was confirmed by immunoblotting with anti-Flag and anti-HA (Cell Signaling) antibodies. Immunoprecipitates were assayed for PI3K activity by measuring phosphorylation of phosphatidylinositol as described in Serunian et al [8], with minor modifications. Briefly, immunoprecipitates were preincubated with sonicated lipids (3 µg phosphatidylinositol and 6 µg phosphatidylserine, Avanti Polar Lipids) at room temperature for 10 min. Enzyme reactions were initiated by the addition of [^γ32^P]ATP (20 µCi, Perkin Elmer), ATP (40 µM final concentration) and MgCl_2_ (10 mM, final concentration) in HEPES buffer (pH 7.4) and incubated at room temperature for 20 min. The reactions were stopped by the addition of HCl (1.3 N final concentration) and the lipids were extracted with MeOH/CHCl_3_ (1∶1 v/v). After mixing vigorously to separate the phases, the organic phase was spotted onto silica gel 60 TLC plates (Merck) that were baked at 100°C before use. Phosphorylated lipids were separated overnight by TLC using an acetic solvent system (n-propanol-2.0M acetic acid [65∶35 v∶v]). TLC plates were exposed to phosphor screens and imaged on the Storm Phosphor Imaging System (Amersham). Spot intensities were quantified using ImageQuant software (Amersham). Levels of phosphorylated lipid product were normalized to P110α protein levels, as determined by western blot.

### Immunoblot analyses

Whole cell extracts were resolved using 4–12% Bis-Tris gradient gels (Invitrogen) and transferred to PVDF membrane (Millipore) before being probed with antibodies targeting total AKT, pAKT (S473), total ERK1/2, pERK1/2 (T202/Y204), P110α, P110β (all from Cell Signaling), or Vinculin (H-10, Santa Cruz).

### Cytotoxicity assay

The parental interleukin-3 (IL-3)–dependent prolymphoid cell line Ba/F3 was maintained in RPMI1640 medium containing 5% fetal bovine serum supplemented with 5 ng/ml IL-3. Ba/F3 cells stably expressing *PIK3R1* mutants by lentiviral-based transduction were maintained in medium without IL-3. These parental or transfected Ba/F3 cells (5×10^3^) were seeded in 96-well plates and treated with DMSO or MK2206 in the presence or absence of IL-3 for 72 hours. The stock solution of MK2206 was prepared in DMSO and used at concentrations ranging from 0.002 to 10 µM. Cell viability was determined using Cell Titer Blue (Promega) for mitochondrial dehydrogenase activity according to the manufacturer's instruction. Two independent experiments, each in triplicate, were performed. Dose-response curves and comparison of fit was performed via four parameter (variable slope) non-linear regression fit of log-normalized data in GraphPad Prism.

### Statistical analyses

Differences in colony formation efficiency were assessed by single factor ANOVA with Bonferroni correction for multiple hypothesis testing.

## Results

In addition to known oncogenic mutations in *PIK3CA*, recent cancer genome sequencing efforts have identified recurrent somatic mutations of *PIK3R1* in GBM, endometrial, and colon cancers [2], [3], [9], [10]. Consistent with these findings, targeted sequencing of 28 human glioma cell lines identified two lines (LN340 and LN428) that carried variant alleles of *PIK3R1* (Table S1). Both mutations target residues (D560 and N564) that were also reported as somatically mutated in primary GBMs [2].

**Figure 1:**
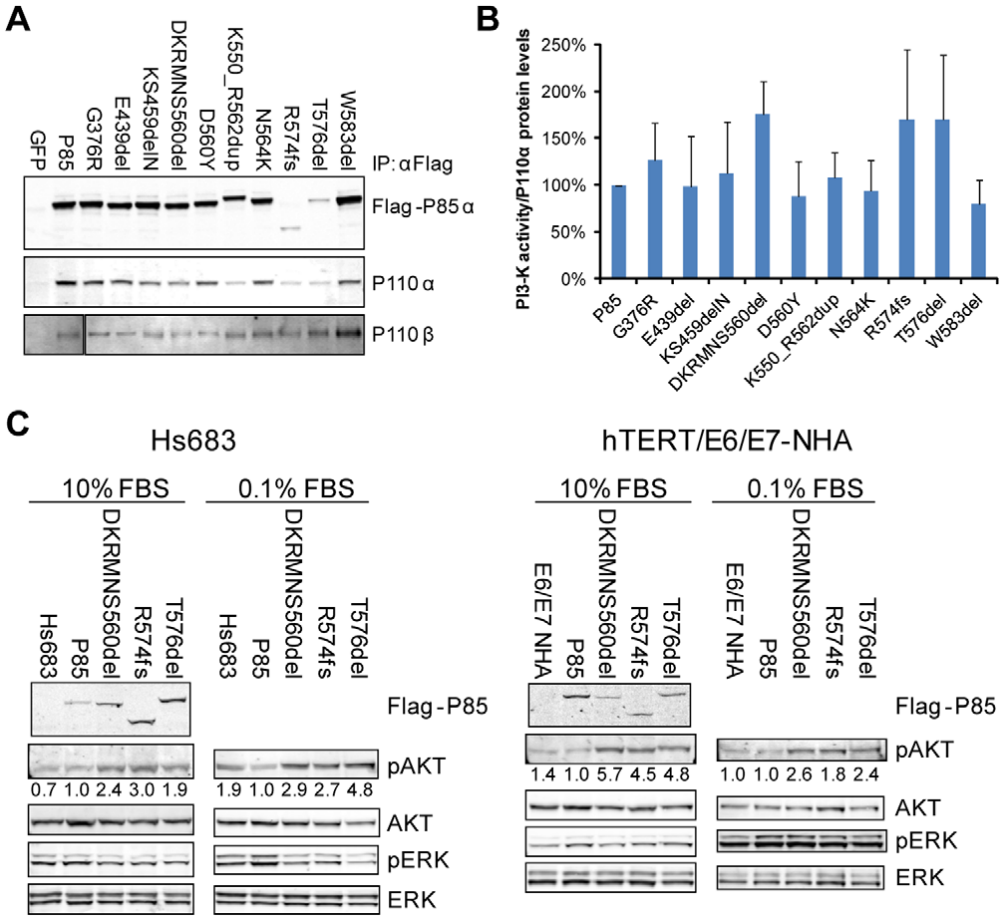
Mutant P85α bound P110α and P110β and increased signaling through the PI3K pathway. (A) GFP, or Flag-tagged wildtype or mutant P85α was expressed in 293T cells and whole cell lysates immunoprecipitated with anti-Flag antibody. Western blotting demonstrated that mutant P85α constructs retained their interaction with both P110α and P110β. (B) Wildtype or mutant P85α was co-expressed in 293T cells with wildtype P110α, and PI3K heterodimers were immunoprecipitated using anti-Flag antibody. *In vitro* kinase activity was assessed by measuring phosphorylation of phosphatidylinositol, and total PIP_3_ signal was quantified and normalized to the total amount of P110α protein loaded in the assay. The average activity from five independent samples is shown (± SD). (C) Expression of mutant P85α constructs increased signaling through the PI3K pathway. Wildtype or mutant P85α was co-expressed with wildtype P110α in Hs683 glioma cells or E6/E7/hTERT-immortalized normal human astrocytes. The resulting cell lines were grown in the indicated concentrations of serum and western blotting was performed to assess activity of the PI3K and MAPK pathways. Representative western blots from at least three experiments are shown. Numerical values below each pAKT panel of the immunoblots represent quantification of the relative protein level by densitometry (normalized to AKT).

As a first step to understand the functional impact of mutations in the nSH2 or iSH2 domains of *PIK3R1*, 10 somatic mutations identified in GBM by TCGA [2] were introduced into a Flag-tagged human P85α construct. Expression of these constructs in 293T cells demonstrated that all mutant forms of P85α retained their ability to interact with the endogenous P110α and P110β catalytic subunits of PI3K (Fig. 1A). An *in vitro* kinase assay confirmed that these mutant P85α/P110α complexes remain catalytically active (Fig. 1B), however, three mutants exhibited enhanced kinase activity compared to wildtype P85α.

To demonstrate pathway activation downstream of increased P110α kinase activity, we selected the three P85α mutants (DKRMNS560del, R574fs, and T576del) with the strongest activities in the *in vitro* kinase assay for biochemical validation in relevant cell systems. Hs683 human glioma cells and E6/E7/hTERT-immortalized normal human astrocytes [11] were co-infected with wildtype or mutant P85α and wildtype P110α expression constructs to achieve stable expression, and AKT phosphorylation was then measured as a downstream readout of PI3K activity. As shown in Figure 1C, increased AKT phosphorylation was observed in cells with enforced expression of mutant P85α under both high and low serum culture conditions, thus confirming that these P85α mutants were able to drive increased signaling through the PI3K pathway independent of upstream, serum-induced, signaling events in GBM-relevant cellular contexts.

**Figure 2:**
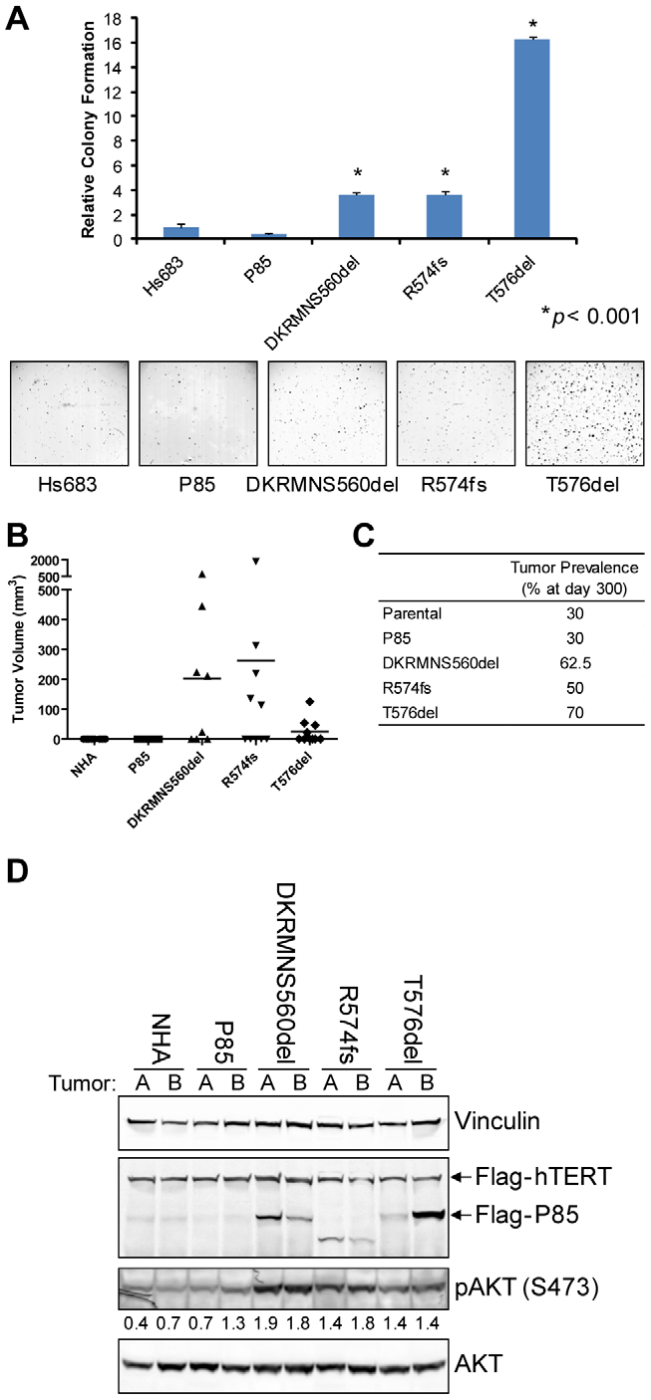
Expression of mutant P85α promoted transformation of GBM-relevant cells both *in vitro* and *in vivo*. (A) Hs683 cells co-expressing wildtype or mutant P85α and wildtype P110α were seeded in soft agar in triplicate. The mean colony numbers (±SD) from a representative experiment are shown. All three mutant P85α constructs significantly (*p*<0.001) promoted colony formation relative to cells expressing wildtype P85α or untransduced cells. (B) E6/E7/hTERT-immortalized normal human astrocytes co-expressing wildtype or mutant P85α and wildtype P110α were injected subcutaneously in nude mice (n = 8 injections for DKRMSNS560del subline, and n = 10 injections for all other sublines). Tumor volume measurements taken 61 days post-transplantation show that P85α mutant tumors were larger. (C) The total penetrance for each subline upon termination of the experiment 300 days post-injection is shown. (D) Whole cell lysates were generated from two tumors of each subline. Western blotting confirmed that tumors retained expression of mutant P85α, and that tumors expressing mutant P85α demonstrated higher levels of pAKT. Numerical values below the pAKT panel of the immunoblot represent quantification of the relative protein level by densitometry (normalized to AKT).

To address the biological relevance of increased kinase activity and enhanced pathway activation by mutant P85α, we next tested their functional activities *in vitro* and *in vivo*. Here, wildtype or mutant P85α constructs were introduced into Hs683 human glioma cells and seeded in soft agar to examine their anchorage-independent growth potential. All three mutant P85α constructs significantly promoted colony formation in this assay of transformation potential (*p*<0.001; Fig. 2A). Furthermore, these mutants enhanced tumorigenicity of human astrocytes *in vivo*. Specifically, wildtype and mutant P85α constructs were stably co-expressed with wildtype P110α in E6/E7/hTERT-immortalized but non-transformed human astrocytes and transplanted subcutaneously in immunocompromised mice (n = 8 injections for DKRMNS560del subline and n = 10 injections for all other sublines). At 61 days post-transplantation, the first analysis time point as defined by the first animal reaching a predetermined experimental endpoint (e.g. tumor reaching the maximum allowable size), an increase in overall tumorigenicity by mutant P85α was readily detected (Fig. 2B). Specifically, 40–50% of the injection sites in the three experimental cohorts that were transplanted with cells expressing mutant P85α had developed measurable tumors, whereas no tumors had formed in the control parental and wildtype P85α cohorts (Fig. 2B). The maximum tumor penetrance of mutant P85α in this model system (62.5% for DKRMNS560del, 50% for R574fs, and 70% for T576del) was achieved by 124 days post-injection (Fig. 2C). Importantly, these mutant P85α-driven tumors were verified to exhibit increased levels of pAKT, indicative of increased flux through the PI3K pathway in these tumors (Fig. 2D). In contrast, when the experiment was terminated at Day 300 post-transplantation, only 30% tumor penetrance was reached in the control parental and wildtype P85α cohorts (Fig. 2C).

**Figure 3:**
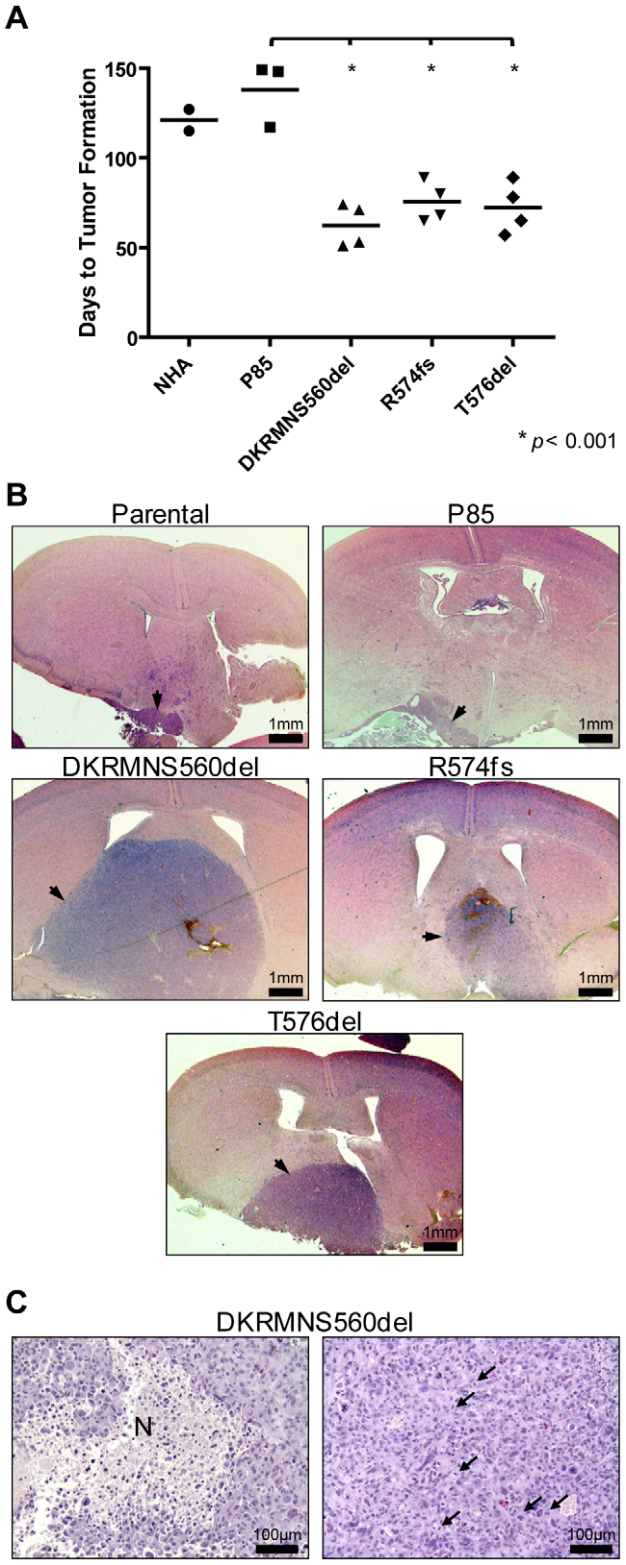
Expression of mutant P85α promoted intracranial tumor formation by primary astrocytes. (A) Scatter plot of the number of days post-injection until neurological or physical symptoms were observed (n = 5 mice were injected in each group). Mutant P85α expressing cells formed more tumors with a significantly shorter latency (*p*<0.001) than cells expressing wildtype P85α. (B) Representative micrographs of hematoxylin and eosin stained tissue sections obtained from mice after intracranial injection of E6/E7/hTERT-immortalized normal human astrocytes co-expressing wildtype or mutant P85α and wildtype P110α. Tumors are indicated by arrowheads, and scale bars represent 1 mm. (C) Representative micrographs showing regions of necrosis (N) and mitotic figures (arrowheads) in an intracranial tumor formed by astrocytes expressing the DKRMNS560del mutant P85α. Scale bars represent 100 µm.

Due to potential limitations of subcutaneous xenografts, we next implanted these same cells at the orthotopic site via intracranial injections. Astrocytes expressing mutant P85α constructs formed tumors significantly more rapidly than astrocytes expressing wildtype P85α (*p*<0.001; median latency was reduced from 148 days to 62–74 days; Fig. 3A). These tumors were also consistently larger with numerous mitoses and evidence of necrosis, which are pathological criteria for the diagnosis of glioblastoma (Fig. 3B, C). However, detailed pathological review of these tumors for evidence of invasion, morphological changes, abnormal mitoses, or vascular changes did not identify compelling trends or differences between tumors expressing mutant P85α relative to the smaller tumors observed in the control cohorts. Together, these results demonstrated that mutations in the iSH2 domain of P85α are oncogenic and capable of promoting transformation of primary immortalized human astrocytes. Furthermore, these experiments confirmed that somatic mutation of *PIK3R1* represents an additional mechanism to deregulate PI3K signaling in developing GBM tumors.

Given the functional significance of these mutations to the establishment and progression of GBM, we next sought to determine if cells expressing mutant P85α proteins are sensitive to a small molecule inhibitor of PI3K signaling. To test this, wildtype, DKRMNS560del, R574fs, or T576del mutant P85α constructs were stably introduced into Ba/F3 cells, which are dependent on exogenous Interleukin 3 (IL-3). Upon introduction of these P85α constructs, Ba/F3 cells were cultured in the presence or absence of IL-3 and exposed to increasing concentrations of MK2206 (synthesized by the Translational Chemistry Core Facility at the M.D. Anderson Cancer Center), an allosteric AKT inhibitor currently undergoing Phase I/II clinical trials [12]. While parental cells, and cells expressing wildtype P85α, did not exhibit differential sensitivity to AKT inhibition in the absence of IL-3, Ba/F3 cells expressing the mutant P85α constructs exhibited significantly increased sensitivity to MK2206 (Table 1). Addition of IL-3 to these cells resulted in decreased sensitivity to the inhibitor, indicating that in the absence of IL-3 the survival of these cells was dependent on AKT activity downstream of mutant P85α. In summary, *PIK3R1* mutations in primary GBM tumor specimens may define a subpopulation of patients who would benefit from treatment with MK2206 or other AKT inhibitors.

**Table 1:** IC50 values (µM) for treatment of Ba/F3 sublines with MK2206.

	Control	IL-3
Parental	0.0232	0.0272
P85	0.0149	0.0146
DKRMNS560del	0.0018	0.0033*
R574fs	0.0026	0.0122**
T576del	0.0037	0.0115**

* *p*<0.05,

** *p*<0.0001.

## Discussion

The PI3K signaling pathway plays a critical role in GBM development and growth [13]. Loss of PTEN is one of the signature genetic alterations of GBM, but other members of the PI3K pathway are also frequently targeted for genetic alteration in GBM [2], [14]. Activation of PI3K signaling through the loss of *PTEN* or via overexpression of AKT has also been shown to promote GBM tumorigenesis in engineered mouse models and primary human cell systems [15]–[17], thus validating the clinical relevance of alterations in this pathway that have been found in GBM patients [18], [19].

In contrast to *PIK3CA*, which is characterized by the predominance of somatic mutations at three “hotspot” residues, somatic mutations of *PIK3R1* in GBM are broadly distributed throughout the nSH2, iSH2, and cSH2 domains of the protein [2], [3], [10]. Sporadic mutations were also identified outside of these three domains, though with decreased frequency [9], [10], [20]. In the current study we focused on the functional effects of three mutations (DKRMNS560del, R574fs, and T576del) that exhibited the highest level of P110α activity in an *in vitro* kinase assay. In particular, we showed that all three mutations promote tumorigenesis *in vivo* in a human GBM-relevant context. This finding is consistent with published studies showing that these three mutations (plus KS459del) potently promoted focus formation in chick embryonic fibroblasts [21], and that both R574fs and T576del mutations were amongst the three most potent P85α mutants for conferring growth factor independence to Ba/F3 cells [10].

Although the other tested mutations of P85α did not directly alter P110α activity in *in vitro* assays, it is conceivable that they may alter PI3K pathway signaling through other mechanisms. For example, one P85α mutant (E160fs) was recently shown to have lost the ability to bind P110α, but still deregulated PI3K signaling through altered binding and stabilization of PTEN [10]. Similarly, a mutation that alters the interaction of the P110α-P85α heterodimer with the lipid bilayer, or of P85α interactions with tyrosine kinase receptors, would not be expected to exhibit increased kinase activity in an *in vitro* setting. For example, the KS459del mutation was shown to increase transformation of chick embryonic fibroblasts [21] while it showed no effect on *in vitro* kinase activity. Thorough mechanistic studies will therefore be required to determine if all observed somatic mutations of *PIK3R1* in GBM are transforming, and if so, by what mechanism(s) they promote tumorigenesis.

Based on current data sets, somatic mutations of *PIK3R1* appear to be limited to a subset of tumor types. While colon cancers, endometrial cancers, and GBM's have been shown to contain a high frequency of *PIK3R1* mutations, sequencing of more than 200 non-small cell lung cancer tumors did not identify mutations in *PIK3R1* [9]. Sequencing of numerous other tumor types has also failed to identify a high frequency of somatic mutations of *PIK3R1*, though the sample sizes in many of these studies remain too small to preclude the existence of *PIK3R1* mutations. Interestingly, ∼45% of *PIK3R1*-mutant colon cancer specimens and ∼22% of *PIK3R1*-mutant endometrioid endometrial cancers also carried mutations of *PIK3CA* [9], [10], [20]. In contrast, mutations in *PIK3R1* were found to be mutually exclusive with mutations in *PIK3CA* in primary GBM tumors [2], [3]. These findings suggest that the overall genetic/developmental context of a tumor dictates the mutational spectrum that will drive tumor development and progression.

While these studies have focused on the role of *PIK3R1* mutations in stimulating PI3K activity, additional studies will have to be performed to determine whether any of these alterations phenocopy the frequent mutations and deletions that are observed in *PTEN*, or whether these independent mutations can act in concert to promote tumor progression. The latter possibility is supported by the common coexistence of mutations in *PIK3R1* and *PTEN* in endometrial cancer [10], and the ability of concurrent mutations in *Pik3ca* and *Pten* to promote the formation of ovarian serous adenocarcinomas in mice [22]. Alone, each of these alterations will likely lead to the upregulation of signaling intermediates such as AKT, but such combinatorial variation of activating mutations may in fact have diverse consequences on parallel signaling networks as well as on the overall state of the cancer cell. It will thus also be necessary to determine how the spectrum of alterations in the PI3K pathway influences the sensitivity of these tumors to inhibitors targeting specific components within the pathway. For example, our results suggest that mutations in the iSH2 domain of *PIK3R1* sensitize GBM cells to the inhibition of AKT by small molecules such as MK2206. However, it is conceivable that in the context of mutation of both *PIK3R1* and *PTEN*, for example, that GBM cells may be sensitive to other inhibitors of the PI3K pathway, or of entirely independent pathways. Given the complexity of these genetic and molecular interactions, it may be necessary to empirically determine how the spectrum of PI3K mutations in a GBM cell impacts its therapeutic dependencies, with the hope of translating these findings to the clinical situation.

In summary, we have demonstrated that beyond biochemical activation of PI3K signaling, somatic mutations of *PIK3R1* are able to promote transformation of primary normal human astrocytes *in vivo*. Taken together, these data support the contention that mutations in P85α, and particularly those in the iSH2 domain, act by allowing P110α to exhibit a higher basal activity level, and thus somatic mutation of *PIK3R1* provides tumors with an additional mechanism to deregulate PI3K signaling and promote tumor progression.

## Supporting Information

Table S1 Results of sequencing the *PIK3R1* and *PIK3CA* loci in 28 human GBM cell lines.
(XLS)
